# 10th European Calcium Society symposium: The Ca^2+^-signaling toolkit in cell function, health and disease

**DOI:** 10.1242/bio.060357

**Published:** 2024-04-25

**Authors:** Femke Speelman-Rooms, Maarten Vanmunster, Aled Coughlan, Macarena Hinrichs, Ilaria Pontisso, Solene Barbeau, Thibaud Parpaite, Geert Bultynck, Malene Brohus

**Affiliations:** ^1^ KU Leuven, Lab. Molecular & Cellular Signaling, Dep. Cellular & Molecular Medicine, Campus Gasthuisberg O/N-I bus 802, Herestraat 49, B-3000 Leuven, Belgium; ^2^ KU Leuven, Lab. Chemical Biology, Dep. Cellular & Molecular Medicine, Campus Gasthuisberg O/N-I bus 901, Herestraat 49, B-3000 Leuven, Belgium; ^3^ Cardiff University, Biomedicine Division, School of Biosciences, Sir Martin Evans Building, Museum Avenue, CF10 3AX, Cardiff, Wales, UK; ^4^ University Medical Center Hamburg-Eppendorf, The Calcium Signalling Group, Department of Biochemistry and Molecular Cell Biology, Hamburg 20251, Germany; ^5^ Institut de Biologie Intégrative de la Cellule (I2BC) - Université Paris-Saclay, Gif-Sur-Yvette, 91190, France; ^6^ UC Louvain, Institute of Neuroscience, Pôle Cellulaire et Moléculaire, avenue Mounier 53, 1200 Brussels, Belgium; ^7^ Aalborg University, Dept. Chemistry and Bioscience, Fredrik Bajers Vej 7H, 9220 Aalborg, Denmark

**Keywords:** Ca^2+^-transport systems, Calcium signaling, Channels, Mitochondria, Endoplasmic reticulum, Molecular physiology, Organellar contact sites

## Abstract

The 10th European Calcium Society symposium, organized in Leuven, Belgium on November 15-17, 2023, focused on the role of Ca^2+^ signaling in cell function, health and disease. The symposium featured six scientific sessions, 16 invited speakers – of whom two were postdoctoral researchers – and 14 short talks. The talks covered various aspects of intracellular Ca^2+^ signaling and its implications in pathology. Each session was opened by one or more invited speakers, followed by a series of presentations from speakers selected from submitted abstracts. Through short talks, poster presentations, awards, and sustainable travel fellowships, the symposium also fostered opportunities for the active participation of early-career researchers. At least half of the short talks were allocated to early-career researchers, thereby offering a platform for the presentation of ongoing work and unpublished results. Presentations were also broadcast in real-time for online attendees. In this Meeting Review, we aim to capture the spirit of the meeting and discuss the main take-home messages that emerged during the symposium.

## Teaser

**Summary:** The Meeting focused on intracellular Ca^2+^ in all its facets. More than 60 researchers discussed their most recent research related to Ca^2+^ stores, Ca^2+^-binding proteins and Ca^2+^-transport systems, the complexities of inter-organellar Ca^2+^ trafficking, the physiological outputs of Ca^2+^, and Ca^2+^ dysregulation underlying pathogenesis.

## Introduction

**Fig. 1.**
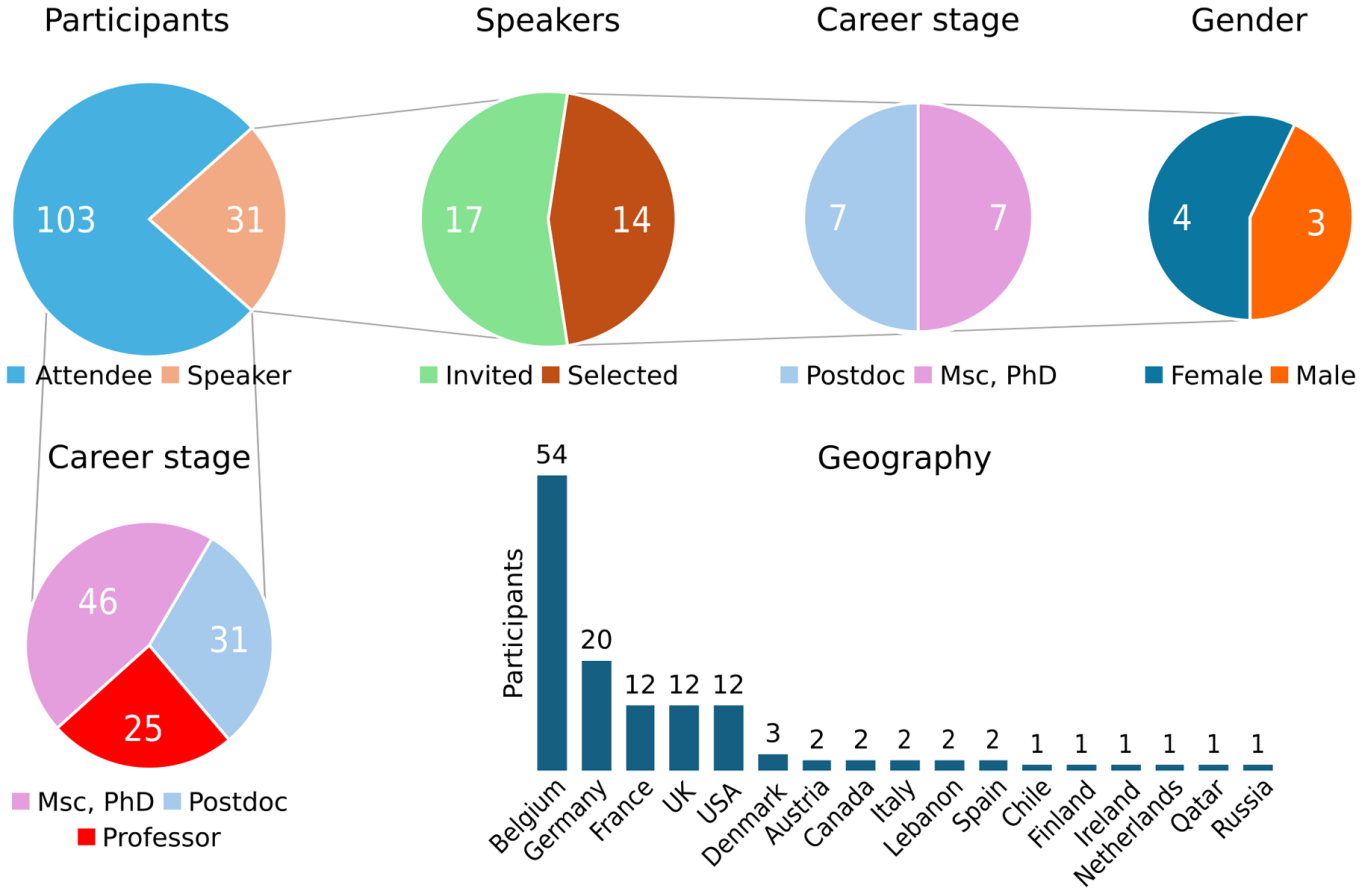
Distribution of attendees and speakers in terms of geography, career stage and gender.

**Fig. 2.**
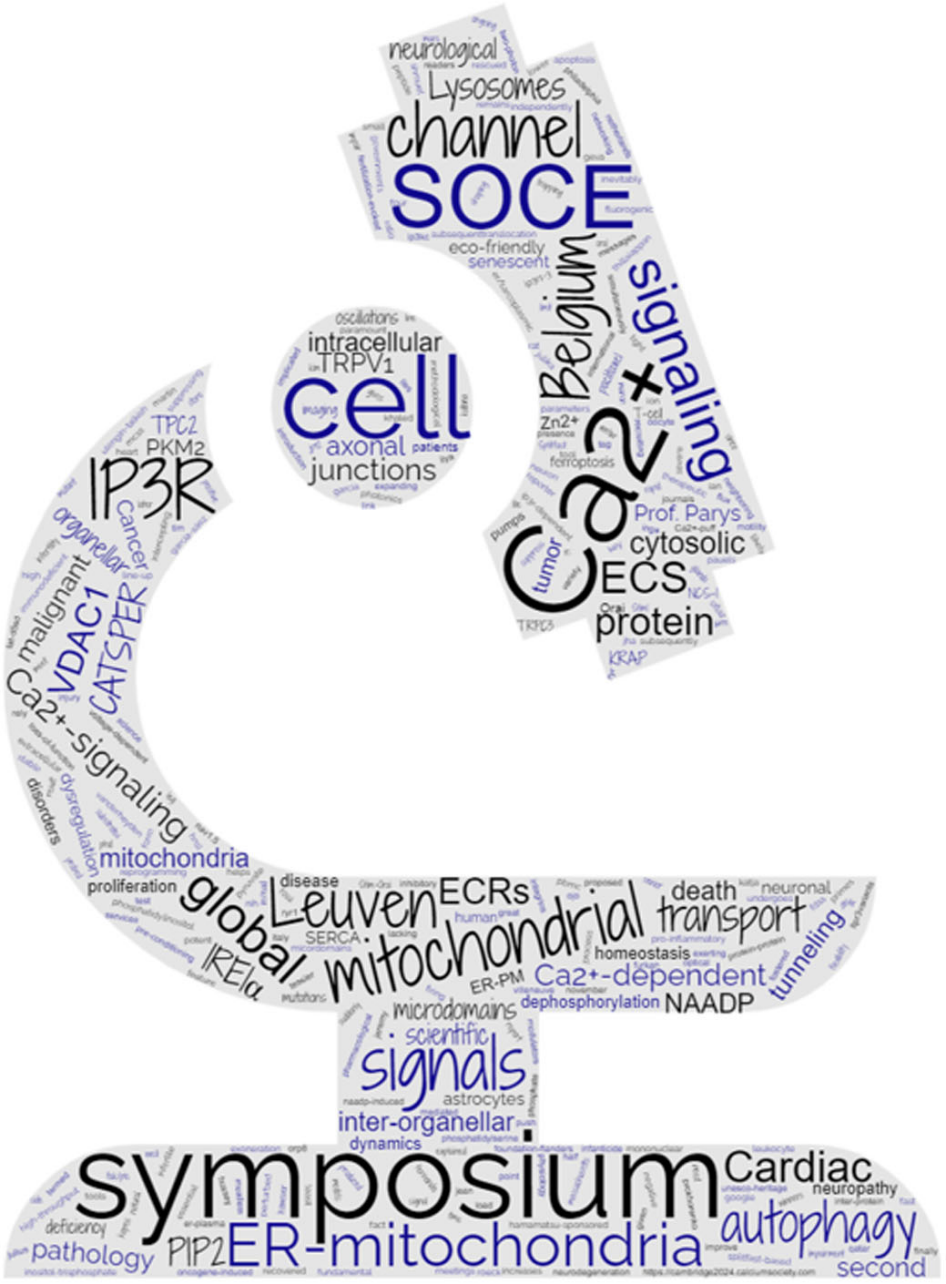
A word cloud representing the different topics of the ECS2023 symposium.

The central theme of the 10th European Calcium Society (ECS) symposium was the Ca^2+^-signaling toolkit in cell function, health, and disease. The meeting was organized at the UNESCO-heritage site Great Beguinage, Leuven, Belgium by Geert Bultynck together with the organizing and scientific committee in honor of the retirement of Prof. Jan B. Parys (KU Leuven, Leuven, Belgium). The symposium commenced with a talk by Jan Parys, reflecting on the evolution of knowledge and techniques in Ca^2+^ research from the early 1980s with prospects for the future. He explored topics such as inositol 1,4,5-trisphosphate receptor (IP_3_R) mobility in the endoplasmic reticulum (ER), membrane contact sites (MCSs), which link the ER to different organelles, Ca^2+^-leak channels, and the role of IP_3_Rs and their protein partners as signaling hubs (Vanderheyden et al., 2009; Lemos et al., 2021; Lemos et al., 2023; Parys and Lemos, 2024). Jan Parys was also an important driving force behind the establishment of the junior ECS (jECS), to promote career opportunities for early-career researchers (ECRs) (Diercks et al., 2021). In that spirit, the 10th ECS symposium enabled the active participation and recognition of ECRs through poster presentations, short talks, awards, and travel fellowships. These fellowships, funded by The Company of Biologists, specifically supported researchers who opted to travel to the conference site using eco-friendly and sustainable travel options. Excitingly, nearly 40% of the 135 participants from around the world were ECRs (Fig. 1). In addition to this, about 12 different online attendees over the 3 days followed the presentations via Google Meet. Below, we provide a brief discussion of the topics covered at the meeting, dealing with intracellular Ca^2+^ in all its facets: Ca^2+^ stores, Ca^2+^-binding proteins and Ca^2+^-transport systems, the complexities of inter-organellar Ca^2+^ trafficking, the physiological outputs of Ca^2+^, and Ca^2+^ dysregulation underlying pathogenesis (Fig. 2).

## Ca^2+^ signaling in organellar contact sites

### ER-mitochondria

Gyorgy Hajnoczky (Thomas Jefferson University, Philadelphia, PA, USA) discussed the localization and function of IP_3_Rs at ER-mitochondrial contact sites. While all three IP_3_R isoforms (IP_3_R1-3) support contact site formation and Ca^2+^ transfer between the ER and mitochondria, IP_3_R2 is the most effective. Moreover, IP_3_R Ca^2+^ release is not required for IP_3_R-dependent ER-mitochondria tethering (Bartok et al., 2019; Katona et al., 2022). Furthermore, rapid and reversible optical trapping of IP_3_Rs at the ER-mitochondria contact sites increased Ca^2+^ signal propagation into the mitochondrial inter-membrane space and matrix (unpublished data, Dr. Hajnoczky).

**Fig. 3.**
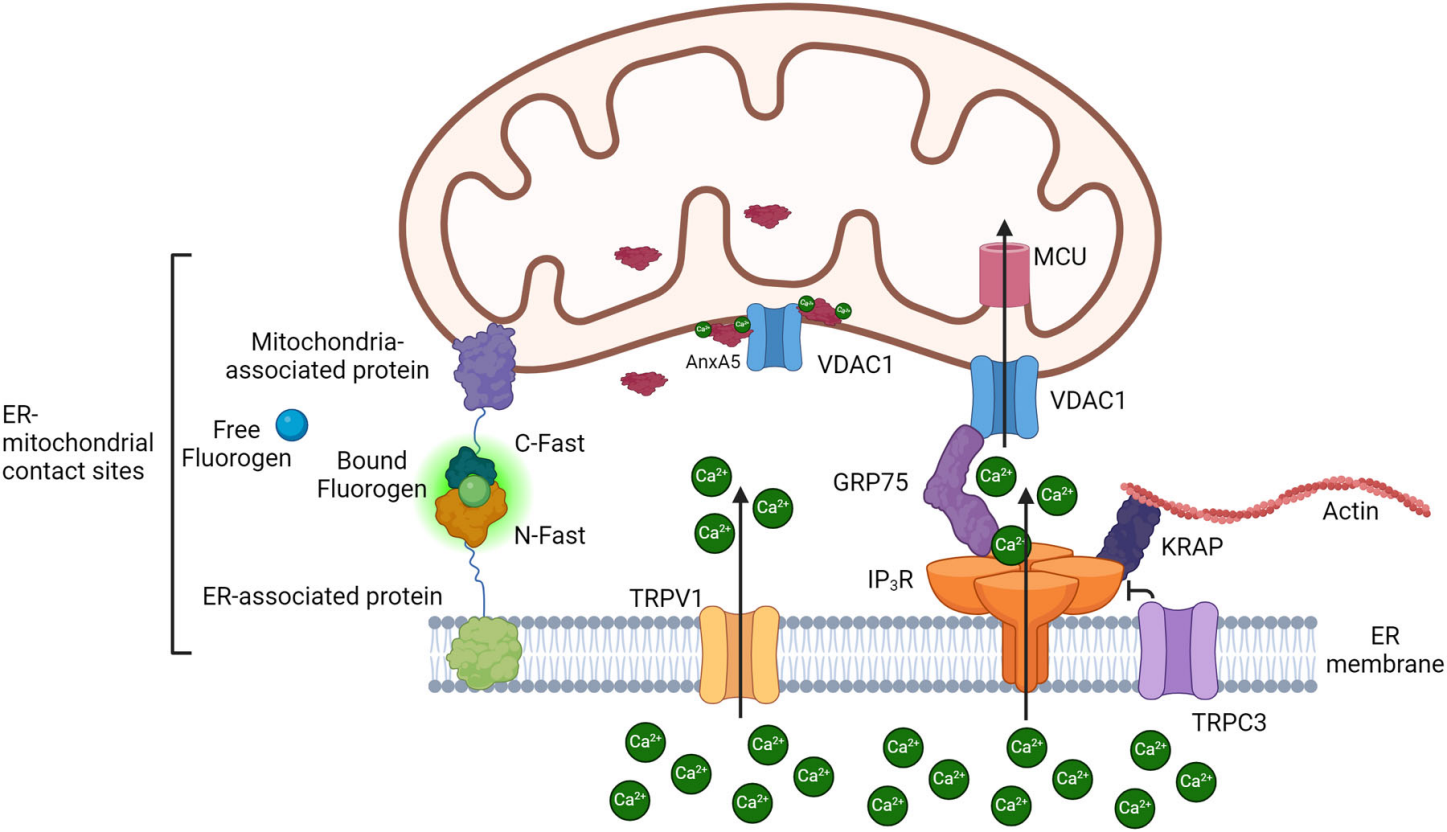
**Scheme of ER-mitochondrial contact sites with resident Ca^2+^-transport systems and their accessory proteins and with the new reporter splitFAST.** IP3R, inositol 1,4,5-trisphosphate receptor; GRP75, 75-kDa glucose-regulated protein; VDAC1, voltage-dependent anion channel 1; MCU, mitochondrial calcium uniporter; TRPC3, Canonical Transient Receptor Potential 3; KRAP, KRas-Induced Actin-Interacting Protein; TRPV1, Transient Receptor Potential Vanilloid 1; AnxA5, Annexin A5. Created on Biorender.

Peace Atakpa-Adaji (Cambridge University, Cambridge, UK) explained how IP_3_Rs are immobilized by KRas-induced actin-interacting protein (KRAP), licensing them to deliver Ca^2+^ from the ER to mitochondria (Thillaiappan et al., 2021). In HeLa cells, IP_3_Rs colocalize with voltage-dependent anion channel 1 (VDAC1) and KRAP at ER-mitochondria junctions (Fig. 3). KRAP knockdown diminishes this association, resulting in loss of cytosolic and mitochondrial Ca^2+^ signals. Hence, KRAP acts a dual regulator at ER-mitochondria junctions by licensing IP_3_Rs to release Ca^2+^ and by regulating the spatial localization of IP_3_Rs at these junctions (unpublished data, Dr. Atapka-Adaji).

Sylvie Ducreux (Université Claude Bernard Lyon, Lyon, France) expanded on mitochondria-associated ER membrane (MAM) components by showing that transient receptor potential vanilloid 1 (TRPV1) channels (Fig. 3) contribute to ER-mitochondrial Ca^2+^-coupling. In a rat cardiomyoblast cell line, TRPV1 channels were found in ER membranes, including some in contact with mitochondria. Acute activation of TRPV1 increased mitochondrial Ca^2+^ levels, thereby advocating for a role for TRPV1 in mitochondrial Ca^2+^ uptake from the ER. Moreover, prolonged/sustained TRPV1 activation decreases MAM interactions, thereby reducing mitochondrial Ca^2+^ accumulation. Furthermore, pharmacological activation of TRPV1 during the pre-conditioning phase of hypoxia/reoxygenation counteracted the associated cell death (Tessier et al., 2023).

A novel assay to monitor real-time dynamics of ER-mitochondria junctions in living cells was presented by Paola Pizzo (University of Padova, Padova, Italy). The assay is based on low affinity variants of the splitFAST reporter. This reporter operates through the fluorescent recomplementation of N-FAST and C-FAST into the fluorogenic reporter FAST (fluorescence-activating and absorption-shifting tag) that is capable of specifically and reversibly binding fluorogens (Tebo and Gautier, 2019) (Fig. 3). As such, this sensor allows the reversible and dynamic detection of protein-protein interactions with broad spectral flexibility. The Pizzo team designed a novel series of splitFAST-based sensors to dynamically study ER-mitochondrial contact sites and their Ca^2+^-signaling properties. The sensors themselves did not evoke the formation of contact sites (García Casas, et al., 2023 preprint). Using such a splitFAST sensor system in HeLa cells, they established that ER-mitochondria interactions are critical for the formation of Ca^2+^ microdomains. It also revealed that the Ca^2+^ content in the ER lumen modulates ER-mitochondria coupling, since a decrease of ER luminal Ca^2+^ increased the organellar interaction, likely involving stromal interaction molecule 1 (STIM1).

### ER-PM

The interplay between lipid dynamics and Ca^2+^ signaling at ER-plasma membrane (PM) junctions was addressed by Shmuel Muallem (NIH, Bethesda, MD, USA). ER-anchored Oxysterol-binding protein-related proteins, ORP5 and ORP8, promote lipid exchange between the PM and ER to control phosphatidylserine levels in these membranes. ORP5 and ORP8 have opposite effects on Ca^2+^ entry via store-operated Ca^2+^ entry (SOCE) by regulating ER-resident STIM1 clustering at ER-PM contact sites (Chung et al., 2023). Moreover, ER-anchored anoctamin 8 (ANO8) interacts with phosphatidylinositol 4,5-bisphosphate (PIP_2_) at the PM to induce assembly of STIM1–Orai1 complexes and recruit other Ca^2+^-signaling proteins at the ER-PM junction (Jha et al., 2019).

Khaled Machaca (Weill Cornell Medical, Doha, Qatar) discussed Ca^2+^ tunneling, a Ca^2+^-signaling modality where Ca^2+^ entering the cell at SOCE microdomains is taken up into the ER by sarcoplasmic/endoplasmic reticulum Ca^2+^ ATPase (SERCA) pumps. These SERCA pumps are localized near ER-PM junctions where STIM-Orai complexes form. Subsequent, Ca^2+^ release occurs via IP_3_Rs located *remotely* from the SOCE puncta and does not result in a global cytosolic Ca^2+^ wave. Interestingly, this mechanism, termed ‘Ca^2+^ tunneling’ through the ER network, allows activation of Ca^2+^-dependent effectors distant from the SOCE site such as opening of Ca^2+^-dependent Cl^−^ channels (Courjaret and Machaca, 2014; Petersen et al., 2017). Inhibition of Ca^2+^ tunneling using a novel molecular construct that inhibits only cortical SERCA pumps near SOCE microdomains, resulted in the lack of Cl^−^ channel activation (unpublished results, Dr. Machaca).

The work of Adelina Ivanova (Cambridge University, Cambridge, UK) revealed that IP_3_Rs located at ER-PM junctions are co-regulated by PIP_2_ and IP_3_, thereby controlling the transition from local to global Ca^2+^ signaling. PIP_2_ partially occupies the IP_3_-binding sites, thereby priming and sensitizing IP_3_Rs to IP_3_. The team combined the overexpression of IP_3_ 3-kinase C (IP_3_KC), intercepting IP_3_ produced from stimulated G-protein-coupled receptors (GPCRs) before it binds to IP_3_Rs, with photo-release of caged IP_3_. This experimental paradigm leads to GPCR activation causing PIP_2_ depletion but not IP_3_R-mediated Ca^2+^ release. Using this approach, it was demonstrated that depletion of PIP_2_ reduced Ca^2+^ puff frequency and delayed local to global Ca^2+^-signal transition (unpublished data, Dr. Ivanova).

### ER-lysosomes

ER-lysosome contact sites are important in regulating Ca^2+^-signaling pathways leading to autophagy or endolysosomal transport. Tim Vervliet (KU Leuven, Leuven, Belgium) revealed that ryanodine receptors (RyRs) act as regulators of autophagy by modulating lysosomal function (Vervliet et al., 2017). He proposed a novel cell biological role for RyRs in the control of lysosomal function as modulators of ER-lysosomal contact sites (unpublished data, Dr. Vervliet).

Local Ca^2+^ release can be initiated by lysosomes and globalized by Ca^2+^ release from the ER/sarcoplasmic reticulum (SR) stores via second messengers; nicotinic acid adenine dinucleotide phosphate (NAADP), IP_3_, and cyclic ADP ribose (cADPR). Yet, the detailed mechanisms underlying this globalization of Ca^2+^ signals remain unclear. Using Ca^2+^ chelators and cell-permeant activators of two pore segment channel 2 (TPC2), Yu Yuan (UC London, London, UK) demonstrated Ca^2+^-dependent coupling of TPC2s with IP_3_Rs. Activation of TPC2 sensitizes local Ca^2+^ signals in response to physiological IP_3_, thereby increasing the potency of agonists to evoke global Ca^2+^ signals. Inter-organellar Ca^2+^ fluxes between lysosomes and ER appear fundamental to the transition of local Ca^2+^ release from lysosomes into an ER release mediated global cytosolic Ca^2+^ rise (Yuan et al., 2022).

## Ca^2+^-binding proteins and effectors

Mutations in the cytosolic Ca^2+^ sensor calmodulin (CaM) lead to life-threatening cardiac arrhythmias in children (Jensen et al., 2018; Hussey et al., 2023; Jensen et al., 2023). Malene Brohus (Aalborg University, Aalborg, Denmark) highlighted a particular CaM mutation (G114R), identified in an Australian woman and two of her four children, all of whom died suddenly at a young age (Brohus et al., 2021). These deaths resulted in the mother being convicted of infanticide in 2003. However, in 2021, functional studies revealed that CaM^G114R^ decreased the Ca^2+^ affinity of CaM and impaired CaM-binding to two critical cardiac Ca^2+^ channels, Ca_V_1.2 and RyR2, resulting in delayed channel closure. Moreover, CaM^G114R^ resulted in impaired binding of CaM to the cardiac sodium channel, Na_V_1.5 (Brohus et al., 2023). These findings advocated for a natural cause of death of the two children affected by the mutation, and eventually led to exoneration and release of the convicted mother in 2023, after 20 years in prison.

Another Ca^2+^-sensing mechanism is provided by the Ca^2+^ sensitive serine/threonine phosphatase calcineurin (CaN), which forms complexes with targets and substrates by binding PxIxIT and LxVP motifs (Ulengin-Talkish and Cyert, 2023). Martha Cyert (Stanford University, Stanford, CA, USA) presented a novel CaN target, C16orf74, a small and highly disordered protein containing an unusual combined LxVPxIxIT motif (unpublished results, Dr. Cyert). One CaN protein entity simultaneously binds two C16orf74 proteins, one via the PxIxIT portion of the motif, and the other via the LxVP portion, with the latter interaction being required for dephosphorylation of C16orf74. Dephosphorylation of C16orf74 is controlled by the ratio of CaN and C16orf74. C16orf74 also recruits CaN to the PM due to palmitoylation of C16orf74. Upon Ca^2+^ binding, CaN dephosphorylates C16orf74, thereby evoking the release of CaN from C16orf74 at the PM. CaN subsequently dephosphorylates other targets. Since C16orf74 is upregulated in cancers, the newly identified CaN/C16orf74 interplay may have possible implications in cancer pathology.

Another Ca^2+^-binding protein is annexin-A5, a Ca^2+^-dependent phospholipid-binding protein involved in regulating Ca^2+^ homeostasis. Furkan E. Oflaz (Medical University of Graz, Graz, Austria) demonstrated that annexin-A5 controls Ca^2+^ signaling at the mitochondrial intermembrane space by regulating the permeability state of VDAC1 (Fig. 3). Additionally, annexin-A5's localization near VDAC1 modulates the oligomeric status of VDAC1 during chemotherapeutic cisplatin-induced cell death, thereby counteracting apoptosis (unpublished results, Dr. Oflaz).

Marek Michalak (University of Alberta, Edmonton, Canada) explored the regulation of inositol-requiring enzyme 1α (IRE1α) signaling by the ER/SR luminal environment (Wang et al., 2019). In response to ER stress, IRE1α undergoes dimerization, autophosphorylation, and RNase domain activation. Deletion of the RNase domain of IRE1α in mouse cardiomyocytes results in altered Ca^2+^ transients and impaired cardiac function (unpublished results, Dr. Michalak). These findings point to a new, non-canonical role for IRE1α in shaping heart function.

## Ca^2+^ signaling in cell death and survival

Rafael Fissore (University of Massachusetts, Amherst, MA, USA) explored the interplay between IP_3_R1-mediated Ca^2+^ oscillations underpinning egg activation in mammals, and the Zn^2+^ levels that exponentially increase during oocyte maturation. Depletion of intracellular Zn^2+^ levels in mouse eggs reduced fertilization-evoked Ca^2+^ oscillations, decreased IP_3_R1 activity, and diminished Ca^2+^ leak; despite a stable ER Ca^2+^ store content and number of IP_3_R1 channels. While supplementation of Zn^2+^ recovered Ca^2+^ oscillations, excessive supplementation reduced IP_3_R1 activity, and terminated Ca^2+^ oscillations. Thus, basal Zn^2+^ concentrations ensure an optimal Ca^2+^ response and IP_3_R1 function upon fertilization (Akizawa et al., 2023).

Sperm cell-specific CatSper ion channels control intracellular Ca^2+^ levels and sperm cell motility. Christoph Brenker (University of Muenster, Muenster, Germany) and colleagues developed a CatSper-activity test to identify infertile males with normal sperm parameters, but defective CatSper function. The research team identified several variations in the *CATSPER* gene resulting in failure of the sperm cells to hyperactivate and penetrate the egg coat. Currently, CatSper loss-of-function represents the most common cause of unexplained male infertility (Young et al., 2024).

Regulated necrosis (RN) encompasses a variety of genetically controlled, highly regulated cell death processes, including ferroptosis. Ana J. García-Sáez (CECAD Research Center, University of Cologne, Cologne, Germany) argued for Ca^2+^ ions as the master regulator of RN. Her team also developed an optogenetic system for controlled ferroptosis via degradation of lipid-reducing protein GPX4. This approach revealed that ferroptosis spreads to neighboring cells with a strong dependency on cell confluence in a cell distance-dependent manner. The formation of pores at the PM, a hallmark of RN, gives rise to Ca^2+^ entry (Roeck et al., 2023 preprint).

## Ca^2+^ signaling in physiology

### The immune system

Andreas Guse and Franziska Möckl (University Medical Centre Hamburg, Hamburg, Germany) highlighted how the second messenger NAADP evokes Ca^2+^ signals that are crucial in T cell activation (Wolf et al., 2015). Their group found that in T cells NAADP binds to hematological and neurological expressed 1-like protein (HN1L)/Jupiter microtubule associated homolog 2 (JPT2) inducing Ca^2+^ release from the ER via RyR1 channels (Roggenkamp et al., 2021). They also presented new technological developments to study NAADP-induced Ca^2+^ signaling in living cells (unpublished data, Dr. Guse and Dr. Möckl).

Mariella Weiß (University Medical Centre Hamburg, Hamburg, Germany) showed that T-cell adhesion to laminin-1 and collagen IV induces the formation of Ca^2+^ microdomains that sensitize mouse T cells to activation. The establishment of these microdomains depend on the binding of laminin-1 or collagen IV to integrins via FAK/PLC activity and through IP_3_Rs. This process facilitates Ca^2+^ entry through STIM-Orai1 coupling with subsequent translocation of the transcription factor NFAT-1 to the nucleus (Weiß et al., 2023).

Inga Pauels (University of Muenster, Muenster, Germany) demonstrated that the endolysosomal Ca^2+^ channel TPC2 regulates post-endolysosomal CD63 transport thus modulating leukocyte adhesion and recruitment in inflammation (Goretzko et al., 2023). Histamine-evoked TPC2 activation induces lysosomal Ca^2+^ release, enhancing CD63 transport to Weibel-Palade bodies (Goretzko et al., 2023). These findings provide a better understanding of leukocyte-endothelium interactions.

### The digestive system

Tight regulation of Ca^2+^ microdomains is crucial in Ca^2+^-dependent physiological responses, such as the secretion of digestive enzymes. Using two-photon imaging on live animals David Yule (University of Rochester, Rochester, NY, USA) presented for the first time the spatiotemporal properties of physiological Ca^2+^-signaling events in mouse salivary glands (Takano and Yule, 2022) and pancreatic acinar cells (Takano et al., 2023). Both parasympathetic stimulation and cholecystokinin induce an increase in Ca^2+^ signals in pancreatic acinar cells whereby increasing stimulus strength results in a transition from local to global Ca^2+^ signals (Takano et al., 2023).

## Ca^2+^ signaling in pathophysiology

### Cancer

Mechanistic insights into the altered balance between pro-tumoral senescence and normal autophagy were probed by Natacha Prevarskaya (Université de Lille, Villeneuve d'Ascq, France). Short transient receptor potential channel 3 (canonical transient receptor potential 3; TRPC3), expressed in stromal fibroblasts, controls mitochondrial Ca^2+^ load via negative regulation of IP_3_R-mediated Ca^2+^ transfer from the ER. Upon senescent stress, including oncogene-induced senescence, TRPC3 becomes downregulated in stromal cells thereby evoking mitochondrial Ca^2+^ overload. Such senescent stromal cells promote tumor growth by secreting pro-inflammatory, tumor-promoting factors. Interestingly, restoring TRPC3 levels in such senescent cells, and thus dampening mitochondrial Ca^2+^ overload, is sufficient to counteract the senescent state (Farfariello et al., 2022).

During tumor transformation, metabolic reprogramming occurs, and most tumor cells display an increased mitochondrial membrane potential, thereby augmenting the clearance of cytosolic Ca^2+^ entering from the extracellular environment. As such, Ca^2+^-dependent inactivation of Orai channels is reduced, thereby leading to enhanced and sustained SOCE. Carlos Villalobos (Spanish National Research Council, Valladolid, Spain) investigated whether the transfer of mitochondria from normal cells to cancer cells (a process called ‘mitoception’) could reverse the enhanced SOCE in colon cancer cells. Tumor cells that received mitochondria from healthy cells displayed decreased SOCE, close to values observed in normal cells, due to normalization of the mitochondrial membrane potential. These results suggest that mitochondria from transformed cells promote SOCE and thus malignant processes downstream of SOCE (unpublished data, Dr. Villalobos).

Another feature of malignant cells is the upregulation of pyruvate kinase M2 (PKM2), which drives malignant cell proliferation. Fernanda Lemos (KU Leuven, Leuven, Belgium) showed that PKM2 interacts with and inhibits IP_3_Rs. In comparison to wild-type cells, cells lacking PKM2 displayed increased agonist-evoked cytosolic Ca^2+^ release. Furthermore, TAT-D5SD, a synthetic IP_3_R1-derived peptide that can displace PKM2 from IP_3_Rs, evoked IP_3_R-mediated Ca^2+^ release and cell death, yet independently of PKM2 (Lemos et al., 2023) Hence, TAT-D5SD appears to act on other IP_3_R-accessory proteins besides PKM2, thereby accounting for TAT-D5SD's impact on Ca^2+^ signaling and cell death.

### Neurological disorders

Ilya Bezprozvanny (UT Southwestern Medical Center at Dallas, Dallas, TX, USA) discussed several mechanisms underlying neurodegeneration, thereby highlighting different novel neuroprotective targets. First, increased levels of ER membrane cholesterol promoted Sigma-1 receptor oligomerization and subsequent stabilization of ER-mitochondrial microdomains, thereby exerting neuroprotective effects (Zhemkov et al., 2021a,b; Kim and Bezprozvanny, 2023). Second, perturbed neuronal autophagy was highlighted as an important factor underlying the neurodegenerative processes in Alzheimer's disease. Interestingly, excessive Ca^2+^ release from the ER via RyR channels is known to suppress autophagic flux (Vervliet 2018). Building on these concepts, novel strategies were presented to limit Ca^2+^ release and to restore neuronal autophagy. Inhibition of Ca^2+^ release from the ER using mice expressing gating-deficient RyR2 channels (Zhang et al., 2023) or using positive allosteric pharmacological modulators of SERCA pumps (Dahl et al., 2023; Rakovskaya et al., 2023) restored autophagy and ameliorated Alzheimer's disease outcomes.

The central role of Ca^2+^ in neuropathic pain was similarly in focus. Using a *Drosophila melanogaster* model of chronic pain, Jeremy Smyth (Uniformed Services University of Health Sciences, Bethesda, MD, USA) demonstrated that leg amputation evoked robust Ca^2+^ signals in astrocytes via STIM-Orai activation. Suppressing both STIM and Orai in astrocytes or using a constitutively active Orai mutant argued that astrocyte SOCE acts as an essential and sufficient signaling response that mediates the transition from acute nerve injury to central sensitization and development of thermal allodynia (Prokhorenko and Smyth, 2023 preprint).

Alterations in Ca^2+^ signaling can lead to chemotherapy-induced peripheral neuropathy (CIPN), a side effect of several chemotherapy regimens, including Paclitaxel. Strategies to prevent CIPN were highlighted by Barbara Ehrlich (Yale University, New Haven, CT, USA). This work revealed a critical role for neuronal calcium sensor-1 (NCS-1), a highly conserved Ca^2+^-binding protein that helps maintain intracellular Ca^2+^ homeostasis and regulates Ca^2+^-dependent signaling pathways. The role of NCS-1 in regulating Ca^2+^ homeostasis depends on a functional interaction with IP_3_R1, facilitating its open probability (Nguyen et al., 2019). Paclitaxel evokes calpain activation with subsequent NCS-1-protein degradation, leading to loss of intracellular Ca^2+^ signaling and ultimately to neuropathy and cognitive impairment. Co-administration of Paclitaxel and a low dose of Li^+^ rescued NCS-1 levels and Ca^2+^ signaling associated with CIPN, without compromising Paclitaxel's therapeutic efficacy against breast tumor growth (Mo et al., 2012).

Tom Venneman (KU Leuven, Leuven, Belgium) explored the relationship between neuronal activity-related Ca^2+^ signaling and axonal mitochondrial transport in neurons (unpublished results, Venneman). Elevated Ca^2+^ levels inhibit axonal mitochondrial transport, as demonstrated via KCl-induced depolarization. It, however, proved impossible to trigger such inhibitory mechanism in ‘non-connecting’ axonal segments. In fact, only mitochondrial transport in axonal segments connected to another neuron was susceptible to inhibition by neuronal activity. Ca^2+^ imaging using the ratiometric indicator Fura-2 revealed that axonal Ca^2+^ concentrations scale with firing frequency in the range of 0.1-1 µM. Instead, the impact of KCl-induced depolarization on mitochondrial transport was associated with cytosolic [Ca^2+^] increases that were almost tenfold higher than those occurring during physiological conditions. Hence, these findings indicate a potent, but localized role for neuronal activity-related Ca^2+^ fluctuations in the regulation of axonal mitochondrial transport.

### IP_3_R deficiency

Beyond Ca^2+^-signaling alterations in pathology, a Hamamatsu-sponsored short talk given by Julius Rönkkö (University of Helsinki, Helsinki, Finland) highlighted the impact of IP_3_R deficiency in human diseases, presenting the generation and characterization of human pluripotent stem cells (iPSCs) that lacked all three IP_3_R isoforms. The experimental results revealed that while IP_3_Rs are important regulators of stem cell metabolism, they are not required for the viability and pluripotency of iPSCs (Rönkkö et al., 2023). As IP_3_Rs are increasingly implicated in human diseases, these cell models will provide a robust tool to study the role of IP_3_Rs in different cell types.

Defects in IP_3_R3, caused by mutations in *ITPR3* identified in patients with immunodeficiency, were found to impair Ca^2+^ signaling after T-cell receptor (TCR) stimulation (Neumann et al., 2023), as presented by Julika Neumann (KU Leuven, Leuven, Belgium). Disrupted Ca^2+^ homeostasis and defects in IP_3_-mediated Ca^2+^ release were shown in fibroblasts and peripheral blood mononuclear cells derived from patients carrying these *ITPR3* mutations. The crippled Ca^2+^-signaling events that arise upon TCR activation led to a severe reduction of T-cell proliferation. While some *ITPR3* variants resulted in reduced Ca^2+^ responses, one *ITPR3* variant displayed a complete loss-of-function, consistent with a more pronounced immunodeficient clinical profile of this patient (Neumann et al., 2023).

## Opportunities for ECRs and symposium ethics

Special discounted registratoin fees for ECRs enhanced inclusivity and the possibility to participate in the symposium, and the Flemish government's OJO initiative facilitated the attendance of PhD students and ECRs from Flemish universities. The Company of Biologists provided funding for the sustainable travel of seven researchers, mainly ECRs, enabling international participation from Germany, the Netherlands, France and the UK via eco-friendly transport. Organizers incentivized active participation of ECRs by providing opportunities to present short talks and posters. Furthermore, the talks were streamed via Google Meet, enabling access to researchers who could not travel to Leuven. In addition, ECRs competed for best presentation awards provided by Cell Calcium and BBA-Molecular Cell Research journals. The winners were Tom Venneman, Franziska Möckl, and Adelina Ivanova for best short talks; and Ian de Ridder, Femke Speelman-Rooms, and Dheeraj Kannancheri Puthooru for best poster presentations. The symposium dinner, open to all participants, and the sociocultural and sports activities encouraged networking between senior researchers and ECRs. Another unique aspect of the symposium was the optional site visit to the Laboratory of Molecular & Cellular Signaling, KU Leuven, allowing participants to familiarize themselves with state-of-the-art high-throughput live cell Ca^2+^- imaging approaches using the FDSS/µCELL instrument (Hamamatsu Photonics, France) demonstrated by Jean Marc D'Angelo.

## Discussion

The symposium underlined that the key to expanding our understanding of physiological Ca^2+^-pathways lies in the detail: The fine-tuning of physiological Ca^2+^ signals through meticulous control and recognition mechanisms. These mechanisms rely on the interactions of organelles and proteins to mobilize Ca^2+^ ions in a highly localized manner to elicit appropriate physiological responses. Another consensus was the paramount importance of unraveling pathophysiological mechanisms of diseases caused by Ca^2+^ dysregulation, such as cancer, neurological, cardiac, and immunological disorders, to improve the outcomes of patients suffering from these diseases. To push the current boundaries of Ca^2+^-signaling research, the symposium emphasized the need for continuous development of sophisticated tools that report on inter-organellar and inter-protein contacts, and on Ca^2+^ fluxes across cell and organellar membranes and between cell compartments with high temporal and spatial resolution. Finally, the future of Ca^2+^ research will also inevitably embrace the integration of AI and AI-based techniques.

## Future ECS events

The ECS will continuously promote the exchange of knowledge, insights, and methodological approaches among researchers from around the globe through the organization of meetings and webinars. Moreover, the Society prioritizes inclusion and thus will also facilitate participation to its events by everyone interested. Through travel fellowships specifically dedicated to ECRs and researchers from emerging countries, and through streaming lectures via a Google Meet platform, the Society strives to lower hurdles for participation to its events and to promote equity, diversity, and inclusion. Later in 2024, the ECS will host its 17th International Meeting in Cambridge, UK (September 1st – 4th, 2024), with Sandip Patel, Martin Bootman, Katja Rietdorf, and Ana Rossi as main organizers. The International ECS meeting is preceded by the 5th junior ECS symposium (August 31st – September 1st, 2024). We invite interested readers to visit https://cambridge2024.calciumsociety.com/. In addition to these upcoming events, the ECS hosts a series of webinars featuring one invited speaker and one short talk selected from submitted abstracts, further coordinated by Femke Speelman-Rooms, Manon Callens and Jens Loncke with support of Malene Brohus. The line-up of speakers and topics is available here.
